# NAFLD in Some Common Endocrine Diseases: Prevalence, Pathophysiology, and Principles of Diagnosis and Management

**DOI:** 10.3390/ijms20112841

**Published:** 2019-06-11

**Authors:** Amedeo Lonardo, Alessandro Mantovani, Simonetta Lugari, Giovanni Targher

**Affiliations:** 1 Operating Unit Internal Medicine–Ospedale Civile di Baggiovara-AOU, 41125 Modena, Italy; 2 Section of Endocrinology, Diabetes and Metabolism, Department of Medicine, University and Azienda Ospedaliera Universitaria Integrata of Verona, 37126 Verona, Italy; alessandro.mantovani@univr.it (A.M.); giovanni.targher@univr.it (G.T.); 3 Department of Biomedical, Metabolic and Neural Science, University of Modena and Reggio Emilia, 41125 Modena, Italy; simonetta.lugari@libero.it

**Keywords:** GH deficiency, hypogonadism, hypothyroidism, PCOS

## Abstract

Secondary nonalcoholic fatty liver disease (NAFLD) defines those complex pathophysiological and clinical consequences that ensue when the liver becomes an ectopic site of lipid storage owing to reasons other than its mutual association with the metabolic syndrome. Disorders affecting gonadal hormones, thyroid hormones, or growth hormones (GH) may cause secondary forms of NAFLD, which exhibit specific pathophysiologic features and, in theory, the possibility to receive an effective treatment. Here, we critically discuss epidemiological and pathophysiological features, as well as principles of diagnosis and management of some common endocrine diseases, such as polycystic ovary syndrome (PCOS), hypothyroidism, hypogonadism, and GH deficiency. Collectively, these forms of NAFLD secondary to specific endocrine derangements may be envisaged as a naturally occurring disease model of NAFLD in humans. Improved understanding of such endocrine secondary forms of NAFLD promises to disclose novel clinical associations and innovative therapeutic approaches, which may potentially be applied also to selected cases of primary NAFLD.

## 1. Introduction

When the liver becomes an ectopic deposit of fatty substrates, which is not one of the physiologic functions of this organ, a cascade of pathophysiological and clinical consequences will ensue. Collectively, these complex metabolic, hepatic, and extra-hepatic consequences are referred to as nonalcoholic fatty liver disease (NAFLD) [1,2]. While the global burden of NAFLD has been elucidated [3,4,5], the pharmacological treatment of NAFLD remains investigational [6,7]. This therapeutic uncertainty partly reflects our incomplete understanding of the ultimate driving forces which promote the development and the progression of NAFLD.

The metabolic syndrome defines a set of cardio-metabolic risk factors, primarily comprising increased visceral adiposity, which amplifies insulin resistance, and predisposes to impaired glucose tolerance or type 2 diabetes, atherogenic dyslipidemia, and arterial hypertension. These metabolic abnormalities tend to cluster in a given patient, so that the presence of each of them strongly predicts the development of other abnormalities over time [8]. Moreover, it is widely appreciated that disorders causing deranged serum concentrations of sex hormones, thyroid hormones, and growth hormones (GH) may also predispose to the development of metabolic syndrome [9,10,11,12,13]. This further substantiates the assertion that visceral adiposity and metabolic syndrome are closely linked with, and in control of, multiple hormonal driving forces.

Primary NAFLD may precede, coexist or follow the occurrence of the metabolic syndrome and its individual features [14]. However, NAFLD may also be secondary to myriads of other causes, such as chronic use of steatogenic drugs, viral infections, parenteral nutrition, hereditary conditions, and surgery [15]. Among these causes of secondary NAFLD, some endocrine diseases [e.g., polycystic ovary syndrome (PCOS), primary hypothyroidism, hypogonadism, and GH deficiency (GHD)] also rank among the first and best characterized risk factors for the development and progression of NAFLD [16,17]. For example, hypothyroidism-induced NAFLD recently raised considerable scientific interest as a distinct disease entity, owing to its strong epidemiological basis and unique pathophysiology; it also serves as a disease model which may help in identifying innovative therapeutic avenues virtually amenable to be extrapolated to manage selected cases of primary NAFLD [18,19].

On these grounds, the present review article briefly discusses epidemiological features, pathophysiological mechanisms, and principles of diagnosis and management of NAFLD in patients with PCOS, primary hypothyroidism, hypogonadism, or GHD. It should be noted that NAFLD is an increasingly prevalent and burdensome liver disease, which is probably overlooked by endocrinologists. Conversely, hepatologists may tend to dismiss underlying endocrine derangements in individuals exhibiting seemingly primary NAFLD. Therefore, our specific aims are to sensitize practicing physicians, clinical endocrinologists, and hepatologists to promptly recognize those forms of NAFLD, which may occur secondary to some common endocrine diseases. In addition, we also aim at prompting researchers to identify these secondary NAFLD forms as a naturally occurring disease model, characterization of which may promote a better understanding of pathophysiological mechanisms and management strategies of all forms of NAFLD, notably including selected cases with primary NAFLD.

## 2. Polycystic Ovary Syndrome

### 2.1. Epidemiology and Diagnosis of PCOS

PCOS is the most common form of anovulatory infertility, which affects as many as up to nearly 10% of women of reproductive age in Europe and worldwide, and may also lead to additional health problems in middle and late adulthood. Indeed, women with PCOS have an increased long-term risk of developing type 2 diabetes, hypertension, and coagulation disorders, as well as increased cardiovascular morbidity and mortality [20,21,22]. In 1935, Stein and Leventhal, two American gynecologists, were first in describing PCOS by reporting a series of patients exhibiting the syndromic tetrad of ovarian cysts, chronic anovulation, obesity, and hirsutism [23].

Based on the 2003 Rotterdam diagnostic criteria [24], PCOS is currently diagnosed by the presence of at least two out of the following three features: clinical and/or biochemical hyperandrogenism, chronic oligo-anovulation, and polycystic ovarian morphology, after exclusion of other endocrine disorders, such as hyperprolactinemia, thyroid dysfunction, late-onset congenital adrenal hyperplasia, or androgen-secreting tumors. It should be pointed out that obesity, which was a component of the early descriptions of the syndrome, is no longer considered a diagnostic feature of PCOS [24,25]. However, affecting up to ~40% of these patients, obesity is indeed a common finding in PCOS. The Rotterdam diagnostic criteria introduced at least three different PCOS phenotypes, subsequently named “*classic*” (i.e., presence of hyperandrogenism and chronic oligo-anovulation, with or without polycystic ovarian morphology), “*ovulatory*” (i.e., presence of hyperandrogenism and polycystic ovarian morphology), and “*normo-androgenic*” (i.e., presence of chronic oligo-anovulation and polycystic ovarian morphology) [24]. As one of the results of this high clinical heterogeneity, women with different phenotypes of PCOS may also differ in terms of co-existent metabolic disorders. In particular, the risk of insulin resistance and metabolic syndrome is greatest in women with PCOS exhibiting the “*classic*” phenotype, intermediate in those with the “*ovulatory*” phenotype, and lowest in those with the “*normo-androgenic*” phenotype [25,26]. Therefore, physicians must be aware that pharmacological approaches to these patients should be personalized by taking into account the specific characteristics of each individual patient with PCOS. Whether or not these individual differences in both insulin resistance/metabolic syndrome and androgen excess may also affect the risk of either having NAFLD at baseline or developing incident NAFLD over time is not entirely clear, although the currently available evidence suggests that this may indeed be the case. 

### 2.2. Epidemiological Evidence Linking PCOS to NAFLD

An ever-increasing number of cross-sectional and case-control studies strongly suggest that the prevalence of NAFLD (in most cases diagnosed with ultrasonography) is remarkably increased in young women with PCOS, regardless of overweight/obesity and other features of the metabolic syndrome (such as extensively reviewed in Reference [27]). In these studies, the prevalence of NAFLD in women with PCOS ranges from approximately 35% to 70% compared to approximately 20% to 30% in age- and body mass index (BMI)-matched control women [27]. Notably, some small case-control studies performed at tertiary gastroenterology centers also documented that PCOS is highly common among young women with biopsy-proven NAFLD [28,29,30]. Indeed, among these patients, the prevalence of PCOS ranged from approximately 50% to 70%, and these women were also more likely to have the most severe histological forms of NAFLD (i.e., nonalcoholic steatohepatitis (NASH), advanced fibrosis, or cirrhosis) [28,29,30]. With regard to the association between PCOS and the histological severity of NAFLD, Setji et al. [28] were first in demonstrating the association of biopsy-proven fibrosing NASH with PCOS in their retrospective chart review study of 200 United States patients with PCOS.

An updated and comprehensive meta-analysis of 17 observational studies, involving a total of 2734 women with PCOS and 2561 healthy women of similar age and BMI, recently confirmed that young women with PCOS had an almost two-fold higher risk of prevalent NAFLD than control women (fixed-effects odds ratio (OR) 2.25, 95% confidence intervals (CI) 1.95–2.60; *I*^2^ = 57%) [31]. Moreover, women with PCOS and hyperandrogenism (i.e., the “classic” phenotype) had a significantly higher risk of prevalent NAFLD, even after adjustment for BMI and other potential confounding factors. Conversely, normo-androgenic women with PCOS did not seem to have a higher prevalence of NAFLD when compared to control women [31]. As reported previously, it is reasonable to believe that this differential impact of the PCOS phenotypes on the risk of developing NAFLD is largely due to the fact that the risk of insulin resistance and metabolic syndrome is greatest among PCOS women with the “*classic*” phenotype, intermediate among those with the “*ovulatory*” phenotype, and lowest among those with the “*normo-androgenic*” phenotype.

Notably, the 13 studies included in the aforementioned meta-analysis, which performed multivariable logistic regression analyses adjusting for age, BMI, serum triglycerides, and insulin resistance, confirmed that increased serum androgen levels (i.e., total testosterone and free androgen index) were independently associated with NAFLD in women with PCOS [31]. Overall, PCOS women with NAFLD also had significantly higher total testosterone levels (mean difference: 0.40 nmol/L, 95% CI 0.29–0.50 nmol/L) and, especially, higher free androgen index (mean difference: 4.46, 95% CI 3.53–5.39) than their counterparts without NAFLD [31]. In line with these findings, in a small case-control study involving young women with PCOS (*n* = 29) and healthy control women (*n* = 22) who were matched for age, BMI, and waist circumference, Jones et al. [32] found that women with PCOS and hyperandrogenism had markedly higher intra-hepatic fat content (detected by magnetic resonance spectroscopy (MRS)) than normo-androgenic PCOS women and healthy controls (mean hepatic fat content: 12.9% vs. 0.6% vs. 1.9%, respectively). These inter-group differences in intra-hepatic fat content remained statistically significant even after adjustment for BMI, visceral adipose tissue, and insulin resistance [32]. In a large case-control study, involving 275 non-obese women with PCOS and 892 non-obese controls, Kim et al. found that women with PCOS had a higher prevalence of ultrasound-diagnosed NAFLD than those without NAFLD (5.5% vs. 2.8%), and that, in women with PCOS, the presence of hyperandrogenemia (i.e., higher free testosterone or free androgen index) was associated with NAFLD, independent of age, BMI, insulin resistance, or glycemic control [33].

Recently, Kumarendran et al. [34], utilizing a large United Kingdom (UK) primary care database, performed a population-based retrospective cohort study which included more than 63,000 women with PCOS and ~121,000 age-, BMI-, and location-matched control women registered between 2000 and 2016. Of interest, these authors were able to show that women with PCOS had an increased incidence of NAFLD (hazard ratio 2.23, 95% CI 1.86–2.66), even after adjustment for age, BMI, and dysglycemia. Further to increased BMI and dysglycemia, these authors also identified hyperandrogenism as an additional risk factor potentially contributing to the development of NAFLD in women with PCOS [34]. Other studies reported higher free androgen index and lower plasma sex-hormone-binding globulin (SHBG) levels in PCOS women with NAFLD than in those without NAFLD [35]. However, other investigators did not find any significant difference in free androgen index or SHBG levels between PCOS women with and without NAFLD [36,37]. Future studies in larger cohorts of carefully characterized PCOS women are needed to better elucidate this issue.

### 2.3. Putative Pathophysiological Mechanisms Linking PCOS with NAFLD

**Figure 1:**
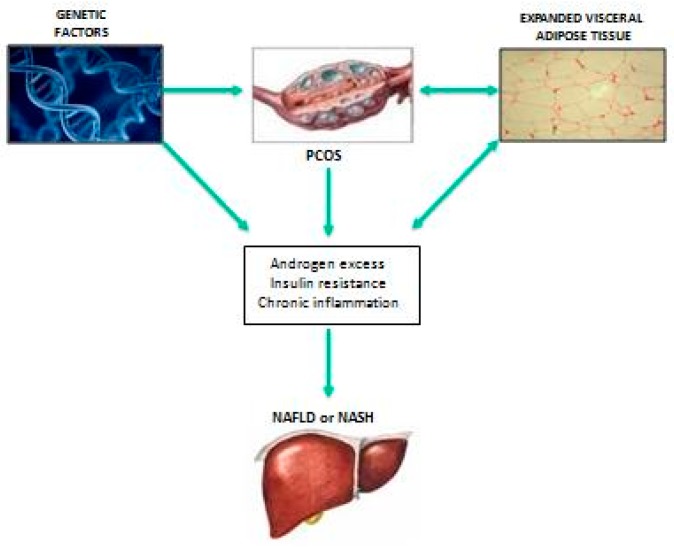
Schematic representation of the putative mechanisms linking polycystic ovary syndrome (PCOS) with the development of nonalcoholic fatty liver disease (NAFLD). NAFLD and nonalcoholic steatohepatitis (NASH) secondary to PCOS are a unique model of endocrine liver disease featuring progressive liver disease in young women with amenorrhea.

A detailed discussion of the putative underlying biological mechanisms through which PCOS may promote the development and progression of NAFLD is beyond the scope of this review article and was more extensively addressed by our group elsewhere [27]. Briefly, there is now an accumulating body of clinical and experimental evidence suggesting that PCOS and NAFLD share common pathogenic mechanisms and are part of a more complex and intriguing network of genetic, clinical, and pathophysiological features (i.e., metabolic, endocrine, and inflammatory abnormalities) [27]. As shown in Figure 1, it is plausible to assume that the pathophysiological mechanisms underpinning the increased risk of NAFLD in PCOS are multi-factorial, involve both genetic and acquired factors, and are likely to represent a complex interplay between abdominal adiposity/overweight/obesity, systemic insulin resistance, chronic inflammation, and hyperandrogenism [27]. Some of these biological actors, such as abdominal adiposity/overweight/obesity and insulin resistance are acknowledged to be major players in the general pathophysiology of NAFLD [27,38]. Experimental studies also supported a key role of insulin in the regulation of ovarian androgen biosynthesis in PCOS. In fact, multiple studies showed that insulin stimulates androgenesis by increasing ovarian androgen production and decreasing hepatic SHBG synthesis. The cellular mechanisms via which insulin regulates androgenesis are not well understood, but potential pathways were proposed [39]. Experimentally, it was demonstrated that insulin acts through its own receptor in ovarian theca cells. Specific blockade of phosphoinositide 3-kinase (PI3K) in normal human theca cells markedly inhibits the combined insulin and luteinizing hormone (LH) stimulation of P450c17 activity [40]. It was also demonstrated that insulin stimulates P450c17 activity through some players of the mitogen-activated protein kinase (MAPK) pathway, such as MAP kinase-activated protein kinase-3 (MKK3/p38) and MAP kinase-activated protein kinase-4/c-Jun N-terminal kinase (MKK4/JNK) [41]. On the other hand, specific inhibition of mitogen-activated protein kinase/extracellular signal-regulated kinase (MEK/ERK), a component of the MAPK insulin pathway, may increase P450c17 activity. Since insulin stimulates MEK/ERK activity, such a defect would not contribute to insulin-stimulated androgen production, but it could promote androgenesis or its responsiveness to stimulation with insulin (via PI3K, MKK3/p38, and MKK4/JNK pathways) and LH/adrenocorticotropic hormone (ACTH) [39,40,41,42]. 

Several studies showed that androgens may induce adipose tissue dysfunction, with adverse effects on lipid metabolism, insulin sensitivity, and adipose tissue expansion [43]. Therefore, androgen-mediated adipose lipotoxicity could represent an important mechanism leading to liver injury in hyperandrogenic PCOS women. Furthermore, in vitro studies also support a direct adverse effect of androgens on hepatic lipid metabolism in women, with testosterone increasing lipogenic gene expression and de novo lipogenesis in human hepatocytes from female, but not male, donors [43]. As discussed below in another section of the article, this sexually dimorphic role of androgens in human metabolic disease is an emerging topic, with female androgen excess and male androgen deficiency sharing an overlapping adverse metabolic phenotype, including abdominal overweight/obesity, dysglycemia, insulin resistance, and NAFLD [43]. However, further studies are needed to better elucidate how hyperandrogenism may directly promote the development and progression of NAFLD in PCOS, and to establish whether treatment with anti-androgenic drugs may reduce the risk of NAFLD in both lean and obese women with PCOS. 

In the last few years, several abnormalities of ovarian angiogenesis with dysregulation of multiple angiogenic factors (including vascular endothelial growth factor, angiopoietins, platelet-derived growth factor, transforming growth factor-β, and basic fibroblast growth factor) were also described in women with PCOS [43]. Thus, it was suggested that this angiogenic factor dysregulation may play a role in PCOS pathophysiology, and might also contribute to ovulatory dysfunction, subfertility, and ovarian hyperstimulation syndrome, which are commonly observed in PCOS women. Based on this fascinating topic, future experimental studies will be needed to explore the potential role of PCOS-related angiogenic factor dysregulation in the development of NAFLD.

### 2.4. Screening and Therapeutic Approaches to NAFLD in PCOS

Given the growing prevalence of NAFLD in young women with PCOS, who are also at risk of developing the most advanced histological forms of NAFLD over time, we firmly believe that the population of young women with PCOS should definitely be considered for systematic screening for NAFLD, especially whenever obesity and clinical/biochemical hyperandrogenism are also present. The optimal method to routinely screen for NAFLD patients with PCOS is currently unclear. However, given the poor sensitivity of serum liver enzyme levels in detecting NAFLD [38], we suggest that, in patients with PCOS, liver ultrasonography and transient elastography (Fibroscan) combined with the use of non-invasive fibrosis markers (e.g., either NAFLD fibrosis score or Fibrosis-4 score) should be used as first-line options for identifying both those individuals with NAFLD and those with suspected NASH to submit to liver biopsy [38]. Moreover, all patients with PCOS should undergo regular follow-up examinations not only for liver-related complications but also for cardio-metabolic diseases [20,21]. 

Currently, although more research is needed to determine the best approach to management of NAFLD in women with PCOS, we propose that a more accurate, patient-centered, multidisciplinary approach to the management and treatment of NAFLD should be considered for young women with PCOS. A first-line therapeutic approach for NAFLD in women with PCOS should focus on lifestyle changes based on hypocaloric diet and increased physical activity [20,25]. A second-line therapeutic approach should consider lifestyle changes plus drug treatment. Metformin remains the drug of choice in PCOS [20,25]. Preliminary evidence also suggests some benefit of pioglitazone in the treatment of women with PCOS [20,44], as well as the use of either liraglutide or other glucagon-like peptide-1 receptor agonists for decreasing intra-hepatic fat content and visceral adipose tissue among women with PCOS who exhibit overweight/obesity [45,46]. However, to date, there is insufficient evidence to either support or refute the use of these newer drugs in women with PCOS and NAFLD. A better understanding of both the complex endocrine regulations of NAFLD and the interconnections linking PCOS with NAFLD will, in our expectations, also result in future advances in the pharmacological treatment of this increasingly prevalent and burdensome liver disease.

## 3. Hypothyroidism

### 3.1. Diagnosis of Primary Hypothyroidism and Epidemiological Evidence Linking Hypothyroidism with NAFLD

Hypothyroidism is a common endocrine disease characterized by thyroid hormone deficiency [47]. Classically, primary overt hypothyroidism is defined as serum thyroid-stimulating hormone (TSH) concentrations above the reference range and free thyroxine (FT4) concentrations below the reference range [47]. Subclinical hypothyroidism is, instead, defined by serum TSH concentrations above the reference range and FT4 concentrations within the normal range [47]. 

The rationale for investigating the association between hypothyroidism and NAFLD is based on two lines of evidence: firstly, the physiologic capacity of thyroid hormones to contrast the development of some features of the metabolic syndrome and, secondly, their direct effects on the liver [48,49,50,51,52]. However, the identification of NAFLD in a given patient with hypothyroidism does not necessarily imply that hypothyroidism is the cause of NAFLD in that specific patient [52]. On these grounds, it can be argued that not all observational studies and meta-analyses published so far clearly demonstrated a causal association between primary (subclinical and overt) hypothyroidism and risk of developing NAFLD, independent of coexisting cardio-metabolic risk factors (Table 1 [53,54,55,56,57,58,59,60,61,62,63,64,65,66,67,68,69,70,71,72,73,74,75,76,77,78,79,80]). For instance, in a large population-based cohort study, involving approximately 9500 Dutch euthyroid individuals from the Rotterdam Study, Bano et al. showed that both subclinical and overt hypothyroidism were significantly associated with an increased incidence of NAFLD, as detected with ultrasonography, even after adjustment for age, sex, BMI, alcohol intake, smoking, lipid profile, hypertension, and diabetes [70]. Similar findings were also reported by Xu et al. in a small prospective case-control study of 327 Chinese patients with subclinical hypothyroidism and 327 age-, sex-, and BMI-matched euthyroid controls [59]. Interestingly, Bril et al. found that, when serum TSH levels were normal, decreasing FT4 levels were significantly associated with increasing intra-hepatic fat content, as assessed by MRS, in a cohort of 232 United States euthyroid patients with type 2 diabetes [69]. However, the authors failed to find significant associations between thyroid function tests and histological features of NAFLD in a subset of patients submitted to liver biopsy [69]. By contrast, in a large longitudinal cohort study of as many as 18,500 South Korean individuals, Lee et al. showed that both subclinical and overt hypothyroidism were not significantly associated with an increased risk of incident NAFLD, as detected by ultrasonography, independent of multiple cardio-metabolic confounders [68]. At present, four published meta-analyses [77,78,79,80] further investigated the relationship between primary (subclinical and overt) hypothyroidism and risk of NAFLD. In the first meta-analysis, including 14 observational studies for a total of nearly 42,000 individuals, Jaruvongvanich et al. showed that NAFLD was not associated with subclinical (random-effects OR 0.63, 95% CI 0.18–2.20), overt (random-effects OR 1.37, 95% CI 0.78–2.41), and unclassified hypothyroidism (random-effects OR 1.21, 95% CI 0.91–1.61) [77]. In contrast, in the subsequent meta-analysis by He et al., including 13 observational studies for a total of approximately 37,000 individuals, both primary subclinical and overt hypothyroidism were associated with an increased risk of prevalent NAFLD, independent of several metabolic confounders (random-effects OR 1.52, 95% CI 1.24–1.87) [78]. Again, in a recent meta-analysis of 12 cross-sectional and three longitudinal studies for a total of 44,140 individuals, both subclinical and overt hypothyroidism were independently associated with an increased risk of prevalent NAFLD (random-effects OR 1.42, 95% CI 1.15–1.77) [79]. However, when the authors meta-analyzed the data from the three longitudinal studies, they found that subclinical hypothyroidism was not associated with an increased risk of incident NAFLD over a median follow-up of five years (random-effects hazard ratio 1.29, 95% CI 0.89–1.86) [79]. More recently, the meta-analysis by Guo et al., including 26 observational studies for a total of 61,548 individuals, documented that patients with NAFLD had significantly higher TSH levels than those without NAFLD, and that unclassified hypothyroidism was significantly associated with an increased risk of prevalent NAFLD [80]. 

With regard to the conflicting results found in observational studies [53,54,55,56,57,58,59,60,61,62,63,64,65,66,67,68,69,70,71,72,73,74,75,76] (Table 1), important differences in terms of study design, population characteristics, definition of hypothyroidism, and diagnosis of NAFLD are the most plausible explanations accounting for variable and inconsistent findings among the published studies. With regard to the meta-analyses [77,78,79,80], the divergent findings may be due to the use of different criteria for inclusion and exclusion of the studies, as well as to the presence of high statistical heterogeneity, which may indirectly reflect the aforementioned differences among the published observational studies. 

Collectively, the currently available data [53,54,55,56,57,58,59,60,61,62,63,64,65,66,67,68,69,70,71,72,73,74,75,76,77,78,79,80] indicate that a significant and independent association between subclinical and overt hypothyroidism and risk of NAFLD remains to be conclusively proven. Large, prospective studies of well-characterized cohorts of patients (i.e., using a clear and consistent diagnostic definition of both hypothyroidism and NAFLD) are eagerly awaited. 

### 3.2. Putative Pathophysiological Mechanisms Linking Hypothyroidism with NAFLD

To date, the biological mechanisms responsible for the development and the progression of NAFLD in individuals with hypothyroidism are incompletely understood. That said, the potential mechanisms linking hypothyroidism to NAFLD include metabolic syndrome, dyslipidemia, insulin resistance, oxidative stress, and direct action of TSH on the hepatocytes [19,49,81]. 

In healthy individuals, the thyroid gland produces two hormones (i.e., thyroxine and triiodothyronine) which, acting through thyroid hormone receptors α and β, play a critical role in cell differentiation during development and help maintain thermogenic and metabolic homeostasis in adults [47].

Individuals with either subclinical or overt hypothyroidism often exhibit features of the metabolic syndrome, such as overweight/obesity, impaired fasting glucose, and atherogenic dyslipidemia [47]. In this context, we highlight that NAFLD typically occurs in close association with impaired glucose and lipid metabolism, as well as perturbed energy homeostasis [14,82], thereby justifying a close connection between hypothyroidism and NAFLD. In fact, as experimentally documented, overweight/obesity per se may blunt the effects of thyroid hormones on the liver, by profoundly affecting glucose and lipid metabolism and energy homeostasis [19,49,81]. In addition, hypothyroidism is also associated with atherogenic dyslipidemia (i.e., hypertriglyceridemia, increased plasma levels of low-density lipoprotein (LDL)-cholesterol and very low-density lipoprotein (VLDL)-cholesterol, reduced plasma levels of high-density lipoprotein (HDL)-cholesterol, and elevated apolipoprotein (apo)B lipoproteins) [82]. Hence, it is possible to speculate that hypothyroidism may further exacerbate NAFLD-associated dyslipidemia, which may also be worsened by insulin resistance [19,81]. 

Hypothyroidism is often associated with insulin resistance [19,51,81]. Preliminary evidence suggests that elevated levels of certain adipocytokines [e.g., leptin, visfatin, tumor necrosis factor-α (TNF-α), and interleukin-1] and increased oxidative stress, occurring during some conditions of hypothyroidism, may concur with the development of insulin resistance [19,49,81]. Alterations in specific cytokine levels and markers of oxidative stress, including reactive oxygen species (ROS) and markers of lipid peroxidation, are shared features which are often observed in patients with hypothyroidism [19,81], as well as in those with NAFLD [82], thus representing a further potential pathophysiological mechanism underpinning the association between hypothyroidism and NAFLD. 

In addition to the adverse effects of decreased serum thyroid hormones on hepatic glucose and lipid metabolism, and potentially explaining the epidemiological finding that also subclinical hypothyroidism may be sufficient to increase the risk of NAFLD, elevated serum TSH per se may promote the development of NAFLD by stimulating hepatic de novo lipogenesis [19,49,81]. The biological activity of TSH is mediated by its specific interaction with the thyrotropin receptor, which is not only expressed on the membrane of thyroid follicular cells, but also in several extra-thyroidal tissues and cells, including the hepatocytes [81]. Interestingly, increasing TSH concentrations may directly induce NAFLD by increasing the intra-hepatocytic triglyceride content [19,81,83]. The molecular mechanism underlying the hepatic steatogenesis induced by increasing TSH levels include activation of hepatic sterol regulatory element-binding transcription factor 1 (SREBP-1c) via the cyclic AMP (cAMP)/protein kinase A (PKA)/peroxisome proliferator-activated receptor-α (PPARα) pathway associated with decreased AMP-activated protein kinase (AMPK), which further increases the expression of genes, finally leading to enhanced steatogenesis [49,81,84]. 

### 3.3. Diagnosis and Management of NAFLD in Patients with Hypothyroidism

Given that hypothyroidism is often associated with features of the metabolic syndrome [19] and that NAFLD is bi-directionally and mutually associated with the metabolic syndrome [79], a strong consideration for NAFLD should be given to patients with hypothyroidism, especially whenever they are overweight or obese [62,79]. However, we highlight that this topic is not covered by guidelines issued by scientific societies; therefore, the aforementioned statement, which is strongly supported by epidemiological evidence, mirrors the opinions of the authors.

Replacement therapy with levothyroxine, the standard treatment of hypothyroidism, is associated with a significant decrease in BMI and serum lipids [47]. To date, there is scarce information on whether treatment of hypothyroidism with levothyroxine is also able to improve NAFLD. In a post hoc analysis of a randomized controlled trial, involving 360 patients with subclinical hypothyroidism, the treatment with levothyroxine proved beneficial on serum liver enzyme levels and ultrasound-diagnosed NAFLD after a 15-month period [85]. In a recent phase IIb single-arm trial, involving 20 euthyroid patients with type 2 diabetes and NAFLD, the administration of low-dose levothyroxine for 16 weeks was associated with a significant reduction of the intra-hepatic fat content, as assessed with MRS [86]. A small multicenter, double-blind, randomized, placebo-controlled phase 2 trial is ongoing to evaluate the efficacy and safety of a highly selective liver-directed, thyroid hormone receptor-β agonist, namely MGL-3196, in patients with biopsy-proven NASH [79]. However, at present, all these data are heavily insufficient to recommend the prescription of levothyroxine in (a subset of) patients with primary NAFLD. Therefore, additional clinical trials with liver histology endpoints are required to firmly establish the potential benefits of the use of either levothyroxine or thyroid hormone receptor agonists in patients with primary NAFLD.

Finally, specific lifestyle modifications (together with replacement therapy with levothyroxine) are strongly recommended for patients with hypothyroidism, NAFLD, and metabolic syndrome features. By contrast, the use of insulin sensitizers and antioxidants was never studied in this subtype of patients and, to date, no available guideline regarding NAFLD or hypothyroidism addresses this emerging aspect.

## 4. Hypogonadism

### 4.1. Definition and Epidemiological Grounds 

Irrespective of its variable underlying pathophysiological mechanisms, hypogonadism defines the presence of reduced sex hormone concentrations in either sex [87]. Thus, while all types of hypogonadism invariably result in low sex hormone levels, the clinical scenario is highly variable as a function of the specific etiology (Table 2) [87]. Despite such a tremendous phenotypic variability, the development and progression of NAFLD are common denominators of hypogonadism, independent of sex and etiology. 

As shown in Table 3, a growing number of studies support a strong and bi-directional association between NAFLD and hypogonadism in both men and women [88,89,90,91,92,93,94,95,96,97,98,99,100,101,102].

With regard to men, patients with NAFLD exhibit reduced serum testosterone levels in cross-sectional studies [89,91], and the lower the testosteronemia, the higher the risk of NAFLD [91]. Moreover, hormone replacement therapy improves NAFLD and features of the metabolic syndrome in hypogonadic men [92]. Finally, preliminary evidence also suggests that men rendered hypogonadal by androgen deprivation therapy for prostate cancer have a higher risk of progressive NAFLD [92].

With regard to women, the prototypic state of female hypogonadism, i.e., the Turner syndrome, is associated with an increased prevalence of raised serum liver enzymes, surrogate indices of NAFLD [93,94,95,96,97,98,99,100]. Moreover, it is known that conditions of estrogen deficiency, which may occur either physiologically in post-menopausal women or in hypogonadism-related diseases, are associated with a higher prevalence of NAFLD and advanced liver fibrosis [101,102]. The risk of advanced hepatic fibrosis is associated with longer duration of estrogen deficiency [102]. 

Post-menopausal status is deemed to be a risk factor for the development and progression of NAFLD in humans [103]. This statement is based on a consistent line of evidence. For example, a case-control study comparing 712 women submitted to oophorectomy to 163 control women found that oophorectomy was an independent predictor of NAFLD [104]. Another large study conducted in 9360 women not only found that the prevalence of NAFLD increased in parallel with increasing age, but also that the prevalence rates of NASH (assessed with a BMI/age/alanine aminotransferase (ALT)/triglyceride (BAAT) index ≥3) were 13.2% vs. 14.9% in women >55 years and obese women with NAFLD, respectively, thus suggesting that post-menopausal age and obesity exert almost the same adverse impact on the risk of developing NASH [105]. At variance with the above studies, in which NAFLD and NASH were diagnosed on a non-invasive basis [106,107], Yang et al., by evaluating data of 541 adults with biopsy-proven NASH, found that men had a higher risk of having more severe liver fibrosis than pre-menopausal women [99]. However, this advantage was lost by women in post-menopausal age whose severity of liver fibrosis was similar to men, suggesting that estrogen protects from hepatic fibrosis. Direct evidence for a beneficial effect of estrogen on the liver was provided by Kamada et al. [107]. These authors reported that ovariectomized mice fed a high-fat and high-cholesterol diet had more severe hepatic histological injury owing to increased intrahepatic infiltration of macrophages, enhanced expression of monocyte chemoattractant protein-1, and increased expression of hepatic inflammatory genes compared to sham-operated controls fed the same diet [107]. Treatment with estrogen was able to improve all the above changes [107]. 

It is known that treatment with the anti-estrogen tamoxifen may increase the risk of NAFLD and NASH in hysterectomized women [94], and that hormonal replacement therapy is associated with a significant improvement in serum liver enzymes in post-menopausal women with type 2 diabetes [95]. Collectively, these findings suggest that estrogens may protect from the development and progression of NAFLD [103,108].

### 4.2. Pathophysiological Mechanisms Linking Hypogonadism with NAFLD

In health states, gonadal hormones play a key role in regulating total body fat and its regional distribution, as well as insulin sensitivity and glucose homeostasis. Conversely, the deficiency of such hormones, such as seen in hypogonadal states, is typically associated with either the full-blown metabolic syndrome or its individual features, notably including the molecular effectors of hepatic steatogenesis, both in experimental disease and in human NAFLD [109].

The biological mechanisms accounting for the association between hypogonadism and NAFLD are very complex and likely to be variable according to the individual categories of hypogonadism. However, some common pathogenic mechanisms may be identified, which closely mirror the biological end-result of the defective hormones (Table 4) [108]. Given that total and regional adiposity (i.e., subcutaneous versus visceral adipose tissue) may critically depend on sex hormones [103], it is clear that hypogonadism will exhibit those features of either overall or visceral obesity, which are known to increase the risk of development and progression of NAFLD via impaired glucose tolerance, hypertension, and atherogenic dyslipidemia [17].

In addition to these common pathophysiological features, some specific mechanisms may also be active in either sex. For example, estrogen deficiency in men results in the development of NAFLD associated with typical features of the metabolic syndrome [110,111]. Moreover, compared to men, women are physiologically more protected from liver injury associated with oxidative stress thanks to the antioxidant properties of estrogen [112]. This accounts for the increased risk of NAFLD and hepatic fibrosis in post-menopausal women. In rat models, the synthesis of fibroblast growth factor (FGF)-21 declines after ovariectomy, thus contributing to the development of NAFLD [113].

Altered gut microbiota may be another potential factor linking acquired hypogonadism with the development of NAFLD in animal models. Harada et al. reported that, in castrated male mice, androgen deprivation was associated with both increased Firmicutes/Bacteroidetes ratio and number of *Lactobacillus* species [114]. Intestinal dysbiosis also exacerbated the cardio-metabolic risk in a sex-dependent manner only in male mice fed a high-fat diet, suggesting that dietary stimuli may amplify cardio-metabolic risk factors via altered gut microbiota [113].

Dehydroepiandrosterone (DHEA), the most abundant steroid hormone, affects insulin resistance, oxidative stress, metabolic homeostasis, and fibrogenesis [115]. Based on this biological background, associated with the observation that NASH follows a rapidly progressive course toward cirrhosis in patients with DHEA deficiency owing to panhypopituitarism, Charlton et al. were first in evaluating serum levels of this hormone in fibrosing NASH [115]. Since this pioneering study, others tried to either confirm or dispute this theory and, of the various studies which, over time, addressed this research question (summarized in Table 5 [90,115,116,117,118,119]), all invariably found that biopsy-proven NASH with fibrosis is associated with reduced serum sulfated DHEA (DHEA-S) levels [105].

### 4.3. Principles of Diagnosis and Management

#### 4.3.1. Diagnosis

The diagnosis of hypogonadism-induced NAFLD requires a high index of suspicion in the individual case. Although this topic is not specifically regulated by current guidelines, the exclusion of other competing causes of (steatogenic) liver disease is a part of the general diagnostic philosophy of NAFLD [38,120,121,122]. On the other side, it seems logical to propose that, in patients with hypogonadism, a first-line non-invasive assessment of the liver should routinely be performed by the endocrinologist in charge of the patient. This may be accomplished by using the same diagnostic approach suggested under Section 2.4 and after exclusion of competing causes of liver disease before referring the patient to the hepatologist whenever fibrosing liver disease is suspected. 

#### 4.3.2. Management

The general principles of treatment include lifestyle changes, namely dietary restrictions coupled with physical exercise, as well as other interventions chiefly aimed at treating comorbid conditions [123].

The specific principles of treatment are usually differentiated on a sexual basis. 

The treatment of choice in *male hypogonadism* is the administration of testosterone rather than aromatase inhibitors, which raise plasma testosterone levels while reducing E2 [123]. Indeed, in men, circulating levels of E2 exceed those of post-menopausal women; estrogen receptors are expressed in many target tissues, and E2 plays a key role in regulating endocrine axes, such as hypothalamic–pituitary–testicular, as well as growth hormone insulin-like growth factor-1, in preserving the reproductive function, bone health, body composition, carbohydrate homeostasis, and vasomotor stability [124]. Since 2015, testosterone products have to be labeled with a warning on potential cardiovascular risk associated with this hormone, despite the fact that this therapy may actually improve such cardiovascular outcomes [125]. 

*Hypogonadism in women* is treated with estrogen replacement through a variety of strategies spanning from treatment with low-dose transdermal E2, oral micronized E2, or per the intramuscular route; after progestin is added, a pill containing both an estrogen and a progestin may be preferable owing to the ease of its use [126]. 

In conclusion, we strongly suggest that patients with hypogonadism-induced NAFLD should be managed jointly by the endocrinologist and the hepatologist with the specific aim of preventing the progression of both liver-related complications and extra-hepatic manifestations of NAFLD [2,127]. The use of sex hormone replacement therapy in selected cases of *primary NAFLD* is an interesting topic of research which requires specific experimental studies and clinical trials, and cannot be recommended at present.

## 5. GH Deficiency

### 5.1. Epidemiology of GH Deficiency

GH and its chief mediator, insulin-like growth factor-1 (IGF-1), are mainly under control of the hypothalamic–pituitary somatotropic axis, even if other elements are involved in their homeostasis and secretion. GH and IGF-1, by regulating glucose and lipid metabolism, body composition, and growth, exert a key metabolic role on the liver, adipose tissue, and skeletal muscle both in children and adults [128,129]. Therefore, GH exerts many effects in different tissues, mediated by complex mechanisms, either directly via interaction with GH receptor or indirectly via its mediator IGF-1 [13]. Specifically, in adult healthy subjects, GH promotes the release of free fatty acids (FFA), via lipolysis in the visceral adipose tissue; moreover; GH also stimulates protein synthesis and exerts anabolic effects on muscle and bone tissue, contributing to an increase in lean body mass, muscle strength, and bone mineral density [130]. The effect of GH and its deficiency on glucose metabolism is quite intricate. It is known that GH stimulates gluconeogenesis and glycogenolysis in the liver and inhibits glucose uptake in adipose tissue, contrasting insulin signaling. Unexpectedly, GHD determines insulin resistance probably due to the increased FFA flux and glycogen synthesis inhibition, but the exact mechanisms remain to be elucidated [129,130].

The actions of the GH–IGF-1 axis are regulated by a complex intracellular signaling pathway, involving Janus kinase 2 (JAK-2) and signal transducer and the activation of activator of transcription 5 (STAT-5), with regulation of target genes leading to an improvement of systemic insulin resistance and lipolysis in visceral adipose tissue [130]. As known, liver is the main tissue involved in IGF-1 production after GH secretion. IGF-1, after binding IGF-binding proteins and its specific receptors (ubiquitously expressed), contributes to systemic metabolic effects, increasing GH action. However, many other extra-hepatic tissues and the liver itself can produce IGF-1, where it exerts autocrine and paracrine effects in a GH-independent manner [131]. Different elements, such as nutritional status, protein and glucose intake, and inflammatory state, are involved in regulating the peripheral and GH-independent production of IGF-1 [132].

GHD in adults (AGHD), in most cases due to either hypothalamic and pituitary lesions or their treatment, leads to detrimental metabolic consequences typically involved in NAFLD pathogenesis, such as visceral obesity, dyslipidemia, and insulin resistance, carrying an inherently increased prevalence of metabolic syndrome [130], an increased risk of premature atherosclerosis, cardiovascular morbidity/mortality, and poor quality of life [128,133]. Based on the finding that untreated AGHD patients exhibit a metabolic syndrome-like phenotype, which is strongly and bi-directionally associated with NAFLD, a potential association between GHD and NAFLD was suggested [134]. 

Confirming this suggestion, both NAFLD and metabolic syndrome are associated with lower serum GH levels and lower GH and IGF-1 levels were consistently reported in NAFLD patients compared to NAFLD-free controls [135,136,137,138,139]. Moreover, lower GH and IGF-1 levels are also associated with the histological severity of NAFLD [139,140,141]. Consistently, in a cohort of patients with biopsy-proven NAFLD, hepatic IGF-1 messenger RNA (mRNA) levels were significantly decreased in patients with NASH compared to those with simple steatosis, and inversely associated with the degrees of hepatic steatosis and inflammation [142]. 

Seen from another perspective, hypothalamic and pituitary disorders, including GHD, are also an increasingly recognized risk factor for the development and progression of secondary forms of NAFLD, which are not reversible following lifestyle changes [143,144,145,146,147,148,149]. Indeed, in the last decades, NAFLD was increasingly diagnosed as a hepatic complication of GHD, carrying a high risk of progressing to more severe forms of NAFLD and requiring liver transplantation (Table 6 [143,144,145,146,147,148]). In a retrospective study, Adams et al. found a prevalence of NAFLD of approximately 2% in patients with hypothalamic or pituitary disorders. Of concern, these patients developed progressive NAFLD, i.e., with histological evidence of either fibrosis or cirrhosis, only a few years after the diagnosis [143]. Specifically, patients with GHD had a 6.4-fold increased prevalence of NAFLD compared to age-, sex-, and BMI-matched controls; in addition, almost all of a selected subgroup of GHD patients had histological evidence of NASH [144]. In agreement, Ichikawa et al. found that, compared to patients with pituitary dysfunction without GHD, those with GHD had a significantly increased risk of NAFLD [145]. In their case-control study, Hong et al. found that the prevalence of NAFLD was significantly higher in men with hypopituitarism than in healthy controls and that, in NAFLD patients, serum GH levels were lower and inversely correlated with the severity of hepatic steatosis [146]. Conflicting with the findings reported above, however, some studies found a similar prevalence of both NAFLD and MRI-assessed hepatic fat content when comparing cohorts of patients with GHD to GHD-free control subjects [149]. Differences in demographic and anthropometric features among the various cohorts might potentially account for these conflicting results [147,148]. The finding that GH replacement therapy induced a small reduction of hepatic fat content in NAFLD patients with GHD [147] offers the reasonable hope to resolve this ongoing scientific controversy by addressing this topic from a therapeutic perspective thanks to adequately sized future large therapeutic trials. 

### 5.2. Pathophysiological Mechanisms Linking GHD with NAFLD 

Based on the metabolic effects of the GH–IGF-1 axis on the adipose tissue and the liver, different pathogenic mechanisms were suggested as a possible link between GHD and NAFLD [150,151], notably including insulin resistance, which is a shared common denominator of both diseases. It was repeatedly observed that mouse models with liver-specific deletion of GH receptor (GHRLD) develop insulin resistance, hepatic steatosis, dyslipidemia, and increased de novo lipogenesis [152,153]. In a cell culture model, Rufinatscha et al. demonstrated a decreased hepatic insulin sensitivity in GHR knockdown hepatocytes, which was not completely reversed by IGF-1 administration, suggesting an important role of GH on hepatic glucose metabolism [142]. The development of NAFLD in liver-specific JAK2-deficient (JAK2L) [154] and liver-specific STAT5-deficient mice [155] highlights the importance of the GH signaling pathway. 

Furthermore, GHRLD mice also exhibit alterations in mitochondrial structure and function and increased oxidative stress with enhanced production of ROS, which in turn further impairs mitochondrial function. Interestingly, these hepatic modifications improve following the administration of either GH or IGF-1, suggesting a GH-independent effect of IGF-1 on the liver [156,157]. In this context, emerging data also suggest that melatonin (a pleiotropic hormone primarily known for its regulatory role in circadian rhythms, sleep, retinal functions, and the immune system) may have a relevant role as a scavenger of ROS with antioxidant properties in individuals with features of the metabolic syndrome [158]. Interestingly, new experimental studies also indicate that, in mouse liver, melatonin is also needed for insulin-stimulated phosphatidylinositol 3-kinase (PI3K)–protein kinase B activity. In rats, it seems to suppress the hepatic glucose production, and, in the human hepatocyte cell line HepG2, it activates glycogen synthesis via a protein kinase C (PKC)ζ–AKT–glycogen synthase kinase-3β (GSK3β) pathway [158]. Moreover, in animal models with high-fat diet (HFD)-induced NAFLD, melatonin seems to be involved in reducing liver and mitochondrial alterations due to oxidative stress and in improving steatosis and histological features [159].

Additionally, a systemic low-grade inflammatory state characterized by increased levels of pro-inflammatory cytokines may also promote the development and progression of NAFLD in GHD patients. In GHRLD mice, Fan et al. showed an increased hepatic expression of fibrogenic and inflammatory genes, such as tumor necrosis-α *(TNF-α)* and C–C motif chemokine ligand 3 *(CCL3)* [152]. In humans, Takahashi et al. found a reduction in TNF-α and C-reactive protein after GH replacement therapy [160]. Another biological activity of GH pertains to its control of hepatocyte proliferation, as demonstrated by GHRLD mice exhibiting impaired hepatocyte regeneration. Instead, IGF-1 seems to affect the biology of hepatic stellate cells by inducing their senescence and inactivation, thereby limiting hepatic fibrogenesis [161].

### 5.3. Principles of Diagnosis and Management

Provocative tests of GH secretion are used to identify GHD, considering the pulsatile GH secretion. The insulin tolerance test, deemed to be the standard test, and the GH-releasing hormone (GHRH)-arginine test, well tolerated, are both used in clinical practice for the diagnosis of GHD. The identification of GH cut-off values for a definite diagnosis of GHD is difficult owing to the lack of normal ranges standardized according to demographic and anthropometric variables. However, a cut-off of 4.1 g/L for the GHRH-arginine test and <5.1 g/L for the insulin tolerance test are considered suggestive for GHD [162,163].

In contrast to pediatric populations, GH replacement therapy should be administrated only in severe and symptomatic forms of AGHD, aiming at reducing visceral fat mass, improving lipid profile, and decreasing cardiovascular morbidity [161]. Despite exerting many positive metabolic effects, GH replacement therapy, however, is not indicated as a possible treatment for NAFLD in AGHD patients [163]. About two decades ago, Takano et al. reported one of the first cases of fatty liver significantly improving after GH replacement therapy in a young patient with pan-hypopituitarism [164]. Subsequently, the role of GH replacement therapy in improving NAFLD in AGHD patients was confirmed [164]. Indeed, GH replacement therapy reduces liver enzymes, and improves hepatic steatosis and biomarkers of inflammation and fibrosis [164,165]. Furthermore, the interruption of GH replacement therapy in adults with childhood onset GHD may increase the risk of developing metabolic complications, including dyslipidemia and NAFLD, indirectly suggesting that long-term GH replacement therapy plays a role in preventing cardio-metabolic risk [166,167]. 

However, considering the negative impact of weight gain on the efficacy of GH replacement therapy [164], it can be hypothesized that other factors may potentially contribute to the progression of NAFLD. Therefore, in GHD adult and pediatric subjects, GH replacement therapy covers a major role in determining an improvement on clinical and histological features of NAFLD, confirming a predominant role of GHD on the development of NAFLD [144,165]. Instead, lifestyle changes merely contribute an additional effect to specific therapy, but they are unable to prevent the progression of liver disease in this type of secondary NAFLD [144,149]. Moreover, patients with GHD often have concomitant derangements of other endocrine pathways, such as thyroid, gonadal, and glucocorticoid hormones, which may all play a major role in the development of secondary NAFLD and NASH via obesity and insulin resistance [132].

In conclusion, based on the evidence reported above, screening for NAFLD seems justified in all patients with GHD. Although data are still controversial, considering its beneficial effects on insulin resistance, inflammation, oxidative stress, and fibrosis, the administration of exogenous GH and especially IGF-1 has a strong rationale and potential clinical applications in the treatment of NAFLD secondary to hypothalamic–pituitary dysfunction(s). Conversely, whether and in which subset of individuals with primary NAFLD hormone replacement therapy with GH/IGF-1 should be tested remains the object of experimental and clinical research.

## 6. Conclusions

NAFLD designates the complex array of metabolic, histological, and extra-hepatic consequences, which result from the intra-hepatic deposition of ectopic fat. As a reflection of our incomplete understanding of its pathogenesis, our capacity to treat NAFLD lags far behind our ever-increasing understanding of the global epidemiological, clinical, and economic burden of disease. 

Given that they are well-characterized forms of secondary NAFLD, PCOS, hypothyroidism, hypogonadism, and GHD were extensively discussed here with specific reference to their epidemiological hallmarks, pathophysiological features, and principles of diagnosis and management.

The findings that both androgen excess (such as observed in certain phenotypes of PCOS) and androgen deficiency (such as seen in male hypogonadism) are associated with an increased risk of NAFLD strongly suggest that the contribution of sex hormones to the development and progression of NAFLD is far from simple. It was logically postulated that metabolic health is associated with a specific androgen/estrogen/androgen ratio in either sex, and changes of this ratio will induce metabolic derangements and NAFLD [168]. In male rodents, androgens, by regulating genes involved in hepatic lipogenesis and glucose metabolism, protect from NAFLD and insulin resistance through a dual mechanism: via androgen receptor signaling and via estrogen receptor signaling (following aromatization of testosterone to estradiol) [169]. In contrast, in female rodents, it is estradiol which protects from NAFLD, whereas androgens promote both NAFLD and dysglycemia [169]. These notions receive credibility by the acknowledged activity of sex hormones and their receptors in regulating liver energy homeostasis and body fat distribution [110]. Stated otherwise, androgen excess in women and androgen deficiency in men both manifest with a shared dysmetabolic phenotype, featuring abdominal obesity, type 2 diabetes, insulin resistance, NAFLD, and excess cardiovascular risk (Table 3). 

We are not aware of any comparative studies specifically addressing similarities and differences between the natural course of NAFLD secondary to endocrine disorders as opposed to the course of primary NAFLD. That said, we point out that the development of hepatocellular carcinoma in individuals with hypothyroidism was reported (reviewed in Reference [19]). This implies that this specific form of NAFLD has the potential to run the complete course of NAFLD from uncomplicated simple steatosis to end-stage liver disease. In contrast, while the presence of advanced fibrosis was consistently reported in patients with PCOS [28], to our knowledge, there are currently no published reports of well-documented cases of hepatocellular carcinoma occurring in patients with PCOS. As far as hypogonadism is concerned, Klinefelter’s syndrome is exceptionally associated with primary biliary cholangitis rather than with NASH–cirrhosis [170,171]. Turner syndrome is rarely associated with NASH–cirrhosis [172]. However, we are not aware of any cases of NAFLD-related hepatocellular carcinoma occurring in hypogonadic patients. 

Collectively, the above-discussed forms of NAFLD secondary to some common endocrine derangements represent a naturally occurring disease model of NAFLD in humans. Therefore, a better understanding of their features promises to disclose novel clinical associations and innovative therapeutic approaches, which may potentially be exploited for use also in selected cases of primary NAFLD. However, further basic and clinical research is certainly needed to achieve this ambitious aim.

## Figures and Tables

**Table 1:** Observational studies and meta-analyses assessing the relationship between (subclinical and overt) hypothyroidism and risk of nonalcoholic fatty liver disease (NAFLD; ordered by publication year and study design).

Ref.	Study Population	Thyroid Function Tests—Definition of Hypothyroidism	NAFLD Diagnosis; Prevalence of NAFLD	Adjustments	Main Findings
**Observational studies**					
[53]	Case-control study: 174 US patients with NASH and 442 age-, sex-, race-, and BMI-matched controls	Hypothyroidism was defined by the self-reported use of levothyroxine	Biopsy	Diabetes, dyslipidemia, hypertension	Hypothyroidism was independently associated with an increased risk of NASH
[54]	Cross-sectional study: 878 Chinese elderly individuals	TSH, FT4, and FT3	USG; 26% with NAFLD	Age, BMI, waist circumference, triglyceride, fasting glucose, uric acid, creatinine	FT4 levels were independently associated with an increased risk of NAFLD
[55]	Cross-sectional study: 1322 Chinese individuals	Hypothyroidism was defined as TSH levels >2.5 mIU/L	USG; 25% with NAFLD	Age, sex, BMI, body fat, triglycerides, systolic blood pressure, diastolic blood pressure, fasting glucose	Hypothyroidism was not independently associated with an increased risk of NAFLD
[56]	Population-based study (Study of Health in Pomerania): 3661 German individuals	Subclinical hypothyroidism was defined as TSH >3 mIU/L and normal FT4; overt hypo thyroidism was defined as TSH >3 mIU/L and FT4 <7 pmol/L	USG; 16% with NAFLD	Age, waist circumference, physical activity, alcohol intake, food intake pattern	Hypothyroidism was not independently associated with an increased risk of NAFLD. Contrariwise, serum FT4 levels were inversely associated with NAFLD
[57]	Case-control study: 246 US patients with biopsy-proven NAFLD and 430 age-, sex-, race-, and BMI-matched controls	Hypothyroidism was defined by the self-reported use of levothyroxine	Biopsy	Alcohol use	Hypothyroidism was independently associated with an increased risk of NAFLD
[58]	Cross-sectional study: 4648 South Korean adults	Subclinical hypothyroidism was defined as TSH ≥4.1 mIU/L and normal FT4; overt hypothyroidism was defined as FT4 <0.7 ng/dL	USG; 25% with NAFLD	Age, sex, BMI, waist circumference, lipids hypertension, diabetes	Subclinical and overt hypothyroidism were independently associated with an increased risk of NAFLD
[59]	Prospective case-control study: 327 Chinese patients with subclinical hypothyroidism and 327 age-, sex-, and BMI-matched euthyroid controls	Subclinical hypothyroidism was defined as TSH ≥4.5 mIU/L and normal FT4 levels	USG; 15% of individuals developed NAFLD over a median follow-up of ~5 years	Waist circumference, systolic blood pressure, diastolic blood pressure, lipids, fasting glucose	Subclinical hypothyroidism was independently associated with an increased risk of NAFLD during the follow-up
[60]	Cross-sectional study: 69 Italian euthyroid patients with biopsy-proven NAFLD	TSH	Biopsy; 44 patients had NASH, whereas 25 patients had only steatosis	Age, sex, BMI, HOMA	TSH levels were independently and positively associated with an increased risk of NASH
[61]	Cross-sectional study: 832 Iranian healthy individuals	Subclinical hypothyroidism was defined as TSH >5.2 mIU/L and normal FT4 levels; overt hypothyroidism was defined as TSH >5.2 mIU/L and FT4 <11.5 pmol/L	USG; 15% with NAFLD	None	Subclinical and overt hypothyroidism were not independently associated with an increased risk of NAFLD
[62]	Cross-sectional study: 753 Mexican adults from the Genetics of Atherosclerotic Disease study	Subclinical hypothyroidism was defined as TSH >4.5 mIU/L and normal FT4 levels	Computed tomography; 31% with NAFLD	None	Subclinical hypothyroidism was not independently associated with an increased risk of NAFLD
[63]	Cross-sectional study: 739 Chinese euthyroid individuals	TSH, FT4	USG; 26% with NAFLD	Age, sex, BMI, smoking status, systolic blood pressure, diastolic blood pressure, lipids, FT3	TSH and FT4 levels were independently associated with an increased risk of NAFLD
[64]	Cross-sectional study: 2576 euthyroid South Koreans	TSH, FT4, FT3	USG; 38% with NAFLD	Age, sex, smoking status, hypertension, fasting glucose, lipids, creatinine, uric acid	FT3 levels, but not TSH and FT4 levels, were independently associated with an increased risk of NAFLD
[65]	Cross-sectional study: 1154 Chinese with chronic hepatitis B	Subclinical hypothyroidism was defined as serum TSH >5.3 mUI/L and normal FT4 levels, whereas overt hypothyroidism was defined as serum FT4 level <7.9 pmol/L and TSH >5.3 mIU/L	Biopsy; 23% with NAFLD	Age, sex	TSH levels were independently and positively associated with an increased risk of NAFLD
[66]	Case-control study: 500 biopsy-proven NAFLD Indians and 300 age-, sex-, and BMI-matched controls	Hypothyroidism was defined by the self-reported use of levothyroxine	Biopsy	Age, sex, BMI, transaminases	Hypothyroidism was independently associated with an increased risk of NAFLD
[67]	Population-based study: 1276 Germans	Subclinical hypothyroidism was defined as TSH ≥3.4 mIU/L and normal FT4 levels; overt hypothyroidism was defined as total T4 levels <12.8 pmol/L and TSH levels ≥3.4 mIU/L	USG; 25% with NAFLD	Age, sex, BMI, waist circumference	Subclinical and overt hypothyroidism were not independently associated with an increased risk of NAFLD
[68]	Longitudinal study: 18,544 healthy South Koreans NAFLD-free at baseline	Subclinical hypothyroidism was defined as TSH >4.2 mIU/L normal FT4 levels; overt hypothyroidism was defined as TSH >4.2 mIU/L and FT4 <0.9 ng/dL	USG; 2348 individuals developed incident NAFLD during a mean follow-up of 4 years	Age, sex, BMI, metabolic syndrome	Subclinical and overt hypothyroidism were not independently associated with an increased risk of NAFLD
[69]	Cross-sectional study: 232 USA euthyroid patients with T2D	FT4	MRS; liver biopsy was performed in patients with a diagnosis of NAFLD; 63% with NAFLD	Age, BMI, hemoglobin A1c	FT4 levels were significantly associated with hepatic triglyceride. However, no associations between thyroid function tests and liver histological parameters (such as inflammation, hepatocyte ballooning, and advanced fibrosis) were observed
[70]	A population-based, prospective cohort study (the Rotterdam study): 9419 Dutch euthyroid individuals	Subclinical hypothyroidism was defined as serum TSH levels >4.0 mIU/L and normal FT4; overt hypothyroidism was defined as serum TSH >4.0 mIU/L and FT4 levels <0.8 ng/dL	Fatty liver index at baseline; USG and Fibroscan_®_ at follow-up; 13% of participants developed incident NAFLD over a median follow-up of 10 years	Age, sex, BMI, alcohol intake, smoking status, follow-up time, use of lipid-lowering agents, lipids, hypertension, diabetes	Compared to euthyroidism, any form of hypothyroidism was associated with an increased risk of NAFLD. Moreover, subclinical and overt hypothyroidism were associated with an increased risk of liver fibrosis as detected by Fibroscan_®_
[71]	Cross-sectional study: 115 Turkish individuals	FT3/FT4 ratio	USG; 60% with NAFLD	Waist circumference, lipids, uric acid, HOMA	FT3/FT4 ratio was independently associated with an increased risk of NAFLD
[72]	Population-based study (Lifelines Cohort Study): 20,289 Dutch euthyroid individuals	TSH, FT4, FT3	FLI (≥60); 21% with NAFLD	Age, sex	FLI ≥60 was independently associated with higher FT3 and lower FT4 levels
[73]	Cross-sectional study: 580 Filipino adults	TSH >4.5 mIU/L without previous history of thyroid disease	USG; 48% with NAFLD	None	NAFLD was not independently associated with TSH levels
[74]	Case-control study 100 Indian adult non-obese hypothyroid patients and 100 age-, sex-, and BMI-matched euthyroid controls	Subclinical hypothyroidism was defined as serum TSH levels ≥4.1 mIU/L and normal FT4 levels, whereas overt hypothyroidism was defined as serum TSH levels ≥4.1 mIU/L and FT4 levels <0.7 ng/dL	USG; 42% with NAFLD	None	NAFLD was diagnosed in 30 patients with hypothyroid and in 12 controls
[75]	Cross-sectional study: 425 South Koreans with biopsy-proven NAFLD	Subclinical hypothyroidism was defined as serum TSH >4.5 mIU/L and normal FT4	Biopsy	Age, sex, BMI, smoking status, hypertension, diabetes, lipids, visceral adipose tissue area, HOMA-IR	Subclinical hypothyroidism was independently associated with an increased risk of NASH and advanced fibrosis
[76]	Population-based study: 3452 Koreans from Korea National Health and Nutrition Examination Survey 2013 to 2015	Subclinical hypothyroidism was defined as a serum TSH levels >6.7 mIU/L with serum FT4 within a normal range	Hepatic steatosis index (≥36)	Age, smoking status, physical activity, income, MetS, waist circumference, lipids, systolic blood pressure, diastolic blood pressure, fasting glucose, urine iodine, thyroid peroxidase antibodies	Subclinical hypothyroidism was independently associated with an increased risk of NAFLD in males, but not in females
**Meta-analyses**					
[77]	14 observational studies for a total of 42,143 individuals	Self-reported history of hypothyroidism with use of levothyroxine replacement therapy or by presence of abnormal thyroid function tests	Imaging and biopsy	Multiple demographic and clinical variables	NAFLD was not independently associated with hypothyroidism
[78]	13 observational studies for a total of 37,194 individuals	Self-reported history of hypothyroidism with use of levothyroxine replacement therapy or by presence of abnormal thyroid function tests	Liver enzymes, imaging, and biopsy	Multiple demographic and clinical variables	Hypothyroidism was independently associated with an increased risk of NAFLD
[79]	12 cross-sectional and 3 longitudinal studies for a total of 44,140 individuals	Self-reported history of hypothyroidism with use of levothyroxine replacement therapy or by presence of abnormal thyroid function tests	Imaging and biopsy	Multiple demographic and clinical variables	Hypothyroidism was independently associated with an increased risk of prevalent NAFLD. Meta-analysis of data from 3 longitudinal studies documented that subclinical hypothyroidism was not independently associated with an increased risk of incident over a median follow-up of 5 years
[80]	26 observational studies for a total of 61,548 individuals	Self-reported history of hypothyroidism with use of levothyroxine replacement therapy or by presence of abnormal thyroid function tests	Imaging, FLI, and biopsy	Multiple demographic and clinical variables	NAFLD patients had significantly higher TSH levels compared to controls. In addition, hypothyroidism was significantly associated with the risk of NAFLD

Abbreviations: BMI, body mass index; FLI, fatty liver index; FT3, free triiodothyronine; FT4, free thyroxine; HOMA, homeostatic model assessment; IR, insulin resistance; NAFLD, nonalcoholic fatty liver disease; MetS, metabolic syndrome; MRS, magnetic resonance spectroscopy; NASH, nonalcoholic steatohepatitis; T2D, type 2 diabetes; TSH, thyroid-stimulating hormone; USA, United States of America, USG, ultrasonography.

**Table 2:** Main causes of hypogonadism (modified from Mintziori et al. [87]).

**Primary** (hypergonadotropic)
Congenital anatomical abnormalities of the gonads
Castration
Specific syndromes (e.g., Turner’s syndrome; Alstrom’s syndrome; Kallmann syndrome)
Drugs (e.g., antiandrogens, antiestrogens, chemotherapy)
Radiation
Hemochromatosis
Autoimmune
**Secondary to pituitary/hypothalamic failure** (hypogonadotropic)
Congenital
Tumor
Trauma
Radiation
Functional

**Table 3:** Epidemiological evidence supporting the notion that patients with hypogonadism are at higher risk of NAFLD.

Ref.	Study Characteristics	Diagnosis of NAFLD	Main Findings
**Hypogonadism in men**			
[88]	A cohort of 117 hypogonadic men (34–69 years), who were treated with HRT (testosterone undecanoate for 1 year)	ALT, AST, and CRP	HRT was associated with a significant reduction in adiposity measures, lipid profile, and rate of individuals who met the criteria of the MetS. Steatosis was strongly associated with all components of the MetS and RLE, which were associated with higher plasma CRP concentrations, significantly decreased following 1-year HRT
[89]	1944 non-drinking men submitted to repeated liver ultrasonography over a median 4.2-year period, with available baseline serum TT level were evaluated	Ultrasonography	Baseline levels of TT were significantly lower in NAFLD individuals in cross-sectional analyses. However, TT level was not independently associated with either development or regression of NAFLD during follow-up
[90]	Retrospective study comparing 75 Chinese IHH men (mean age 21.4 ± 3.8 years, range 17–30 years) to 135 age- and sex-matched healthy controls	Ultrasonography	Compared to healthy controls, IHH men had higher serum ACTH levels, lower cortisol levels, deranged glycolipidic profile and a higher prevalence of NAFLD. NAFLD was independently associated with ACTH levels
[91]	55 consecutive men with chronic SCI admitted to a rehabilitation program were submitted to clinical/biochemical evaluations and liver ultrasonography	Ultrasonography	NAFLD was diagnosed in 49% of cases. TT and FT levels were independently associated with NAFLD; the risk of NAFLD increased by 1% for each decrement of 1 ng/dL of TT and of 3% for each decrement of 1 pg/mL of FT
[92]	Out of 380,669 men with histologically proven prostate cancer, 31,117 elderly men who received ADT were identified by using a representative cancer registry. Individuals with metastatic disease, pre-existent MetS, diabetes, preexisting liver disease, or a history of alcoholism/ alcohol related disorders were excluded	Diagnostic and procedural codes from physician office or inpatient visits	Elderly men submitted to ADT were more likely to be diagnosed with NAFLD, cirrhosis, liver necrosis, and any liver disease
**Hypogonadism in women**			
[93]	Retrospective analysis of serum liver enzymes in 80 women with TS, followed by a prospective study in 20 women with TS following 3 months on-and-off HRT	GGT, AST, and ALP	44% of women with TS had RLE. HRT resulted in a significant reduction in serum liver enzymes without improving serum protein concentrations
[94]	Prospective, double-blind, RCT vs. placebo recruiting 5408 women who underwent hysterectomies and enrolled into the multicentric Italian tamoxifen chemoprevention trial	NAFLD was suspected based on de novo incident chronic unexplained hyper-transaminasemia (× 1.5 n.v.) over a 6-month period, in the absence of competing etiologies and confirmed with ultrasonography. NAFLD women defined as above were offered confirmative liver biopsy	52 out of 64 women who met the predefined criteria in the course of follow-up developed suspected US-confirmed NAFLD: 34 on tamoxifen vs. 18 on placebo, HR = 2.0 (95% CI 1.1–3.5; *p* = 0.04). Further to tamoxifen, overweight (HR 2.4, 95% CI 1.2–4.8), obesity (HR 3.6, 95%CI 1.7–7.6), hypercholesterolemia (HR 3.4, 9%% CI 1.4–7.8), and hypertension (HR 2.0, 95% CI 1.0–3.8) were associated with increased risk of incident NAFLD. Out of 20 women submitted to LB, 15 had mild-to-moderate NASH (12 tamoxifen vs. 3 placebo), and 5 had simple steatosis (1 tamoxifen vs. 4 placebo). None developed cirrhosis over a mean 8.7-year follow-up
[95]	50 women with T2D entered a double-blind, RCT of HRT vs. placebo	AST, ALT, GGT, and ALP	Compared to those randomized to placebo, women randomized and compliant to HRT (*n* = 19) had significant reductions in serum liver enzymes
[96]	Serum liver enzymes were assessed in 169 women (14 with TS and 11 controls with hypogonadism) on HRT with oral E2	GGT, ALT, ALP, albumin, and bilirubin	The prevalence and incidence rates of RLE among women with TS were 91% and 2.1% per year, respectively. RLE were associated with total cholesterol and BMI, and reversed by increasing doses of HRT
[97]	218 women with TS (mean age 33 ± 13, range 16–71 years) from outpatient clinics at Swedish university hospitals.	AST, ALT, ALP, GGT, serology for viral hepatitis and liver-specific auto-antibodies	36% of TS women had one or more RLE; the most prevalent was serum GGT level, which was independently associated with total cholesterol both at baseline and at 5 years. Liver histology findings in 6 TS women submitted to LB included cholangitis (*n* = 1), hepatitis C (*n* = 1), steatosis (*n* = 2), and normal (*n* = 2). Withdrawal of estrogen substitution did not influence serum liver enzymes
[98]	This Mexican cross-sectional study compared anthropometric, metabolic, hormonal (serum estradiol and cortisol concentrations), and biochemical variables in 93 women with NAFLD and as many NAFLD-free controls	Ultrasonography and transient elastography; APRI, NFS	The prevalence of NAFLD in premenopausal, post-menopausal, and PCOS patients was 32.2, 57.9, and 62%, respectively. Age, adiposity measures, fasting glucose, HOMA-IR, and insulin were significantly higher in NAFLD patients. Compared to NAFLD women, those NAFLD-free women had significantly higher levels of serum E2
[99]	541 individuals with biopsy-proven NASH were recruited. LRA was used to determine the association among sex, menopause, and severity of hepatic fibrosis	Liver biopsy	Compared to pre-menopausal women, men and post-menopausal women had a higher risk of advanced liver fibrosis suggesting that estrogens may protect from fibrosis
[100]	Survey of questionnaires referring to 492 patients with TS (age 17.1–42.5 years) administered by attending physicians throughout Japan. For comparison purposes, data from the National Health and Nutrition Survey were used	Liver dysfunction was defined as AST ≥41 U/L, ALT ≥36 U/L, or GGT ≥60 U/L	Women with TS tend to become obese from young ages (15–39 years). Compared to the Japanese general female population, women with TS had higher prevalence rates of diabetes, hypertension, dyslipidemia, and liver dysfunction which were associated with increasing BMI rather than karyotypes
[101]	Retrospective analysis of incident fatty liver and/or RLE during SERM treatment in 1061 women who were treated for breast cancer	Imaging and/or ALT	SERM treatment was independently associated with an increased the risk of incident raised serum ALT levels and/or fatty liver. Consistently, SERM discontinuation was associated with normalization of raised ALT levels in virtually all cases. No cases of liver-related death/ progression to cirrhosis were registered
[102]	Analysis of data from 488 post-menopausal women with histologically proven NAFLD and self-reported information on age at menopause	Liver histology	In post-menopausal women with NAFLD, the duration of estrogen deficiency affected risk of liver fibrosis.

Abbreviations: ADT, androgen deprivation therapy; ALP, alkaline phosphatase; ALT, alanine aminotransferase; APRI, AST-to-platelet ratio index; AST, aspartate aminotransferase; BMI, body mass index; CI, confidence intervals; CRP, C-reactive protein; E2, 17-β-estradiol; FL, fatty liver; FT, free testosterone; GGT, γ-glutamyl transferase; HR, hazards ratio; HRT, hormone replacement therapy; HWR, hip-to-waist ratio; IHH, idiopathic hypogonadotropic hypogonadism; LB, liver biopsy; LRA, logistic regression analysis; MetS, metabolic syndrome; NAFLD, nonalcoholic fatty liver disease; NFS, NAFLD fibrosis score; RCT, randomized controlled trial; RLE, raised liver enzymes; SCI, spinal cord injury; SERM, selective estrogen receptor modulator; TS, Turner syndrome; TT, total testosterone; US, ultrasound.

**Table 4:** Metabolic effects of sex hormones (modified from Shen et al. [108]).

	Glucose Metabolism		Lipid Metabolism	
**Estrogen**	↑ Insulin clearance	↑ Glycogen storage	↑ Lipolysis	↑ Cholesterol removal
	↓Gluconeogenesis		↓Lipogenesis ↓Lipid uptake	↓Cholesterol synthesis
**Androgen**	↑ Insulin receptor	↑ Glycogen synthesis	↑ Cholesterol uptake	↑ Cholesterol synthesis
	↓Glucose uptake	↓Lipogenesis	↓Cholesterol removal	

**Table 5:** Evidence for an association of low serum levels of sulfated dehydroepiandrosterone (DHEA-S) with histologically proven fibrosing NASH.

Ref.	Method	Findings	Comment
[115]	Overall, 439 patients with biopsy-proven mild/advanced NAFLD were enrolled in the USA Simple steatosis and NASH with initial fibrosis (i.e., stages 0–2) were categorized together as “mild NAFLD” NASH with fibrosis stage 3–4 was defined as “advanced NAFLD” DHEA-S was measured with ELISA.	Compared to patients with mild disease, individuals with advanced NAFLD exhibited lower serum concentrations of DHEA-S Decreasing DHEA-S paralleled increasing stages of fibrosis All patients who had advanced NAFLD exhibited as low DHEA-S levels which were compatible with adrenal insufficiency in the majority of cases	NASH with advanced stages of fibrosis is strongly associated with low serum concentrations of DHEA-S
[116]	Data from 133 Japanese patients with biopsy-proven NAFLD (90 with NASH: 73 patients had fibrosis stage 0–2, and 17 had advanced disease as defined by fibrosis stage 3 or 4) were compared to 399 sex- and age-matched healthy controls DHEA-S was measured by CEI	DHEA-S serum concentrations were not statistically different from those found in the controls Lower DHEA-S paralleled with increasing stages of fibrosis and this association remained after adjustment for confounding factors (age, sex, and IR)	Low circulating DHEA-S could have a causal role in the progression of NAFLD to fibrosing NASH
[117]	69 Japanese men who had NAFLD diagnosed with ultrasonography were extracted from a larger sample of 158 Japanese men who had neither viral liver diseases nor an alcoholic intake >20 g/day DHEA-S was measured by RIA	At multivariate regression analysis, serum ALT was positively correlated with serum DHEA-S, serum triglyceride, and BMI	DHEAS levels are increased in patients with NAFLD with elevated ALT levels
[118]	160 individuals with morbid obesity were submitted to liver biopsy which showed SS in 72, NASH with fibrosis stage 0–1 in 60, and NASH with fibrosis stage ≥2 in 12 patients. 16 had normal liver histology DHEA-S was measured by ELISA	With one exception, all patients with NASH and fibrosis stage 2–3 had low serum DHEA concentrations, i.e., <123 μg/dL.	Low serum levels of DHEA are very common among morbidly obese individuals with NASH and advanced fibrosis
[119]	This study enrolled (a) a training cohort of 44 patients with biopsy proven NAFLD and (b) a validation cohort comprising 105 patients with biopsy-proven NAFLD and 26 with biopsy-proven PBC patients. Moreover, 48 age-matched healthy controls who had normal liver tests and no infection with hepatitis viruses were evaluated Metabolites with LMW were identified with capillary electrophoresis and liquid chromatography with mass spectrometry	In the training cohort, increasing severity of hepatic fibrosis was associated with a decrease of DHEA-S and etiocholanolone-S and an increase of 16-OH-DHEA-S. In the validation cohort, the 16-OH-DHEA-S/DHEA-S ratio and 16-OH-DHEA-S/etiocholanolone-S ratio were also strongly associated with the stage of fibrosis. Conversely, DHEA-S, etiocholanolone-S, and the two ratios were not associated with the stage of fibrosis in patients with PBC	Disturbances in the hormonal profile are a specific feature of NAFLD, which could be exploited for therapeutic purposes
[90]	Retrospective case-control study enrolling 75 Chinese men with IHH and 135 age- and sex-matched healthy controls. The diagnosis of presence/absence of NAFLD was based on ultrasonography to which only 63 individuals with IHH were submitted DHEA-S was measured by chemiluminescence	DHEA-S serum concentrations were significantly more elevated among those 22 patients with IHH and NAFLD than among those 41 with IHH without NAFLD	There is a complex interaction among the HPA axis, testosterone deficiency, and perturbed metabolic health in men with IHH

Abbreviation: CEI, chemiluminescent enzyme immunoassay; DHEA-S, sulfated dehydroepiandrosterone; ELISA, enzyme-linked immunosorbent assay; Etiocholanolone-S, 5α-androstan-3β ol-17-one sulfate; HPA, hypothalamic–pituitary–adrenal; IHH, idiopathic hypogonadotropic hypogonadism; IR, insulin resistance; LMW, low molecular weight; NASH, nonalcoholic steatohepatitis; 16-OH-DHEA-S, hydroxy-dehydroepiandrosterone sulfate; PBC, primary biliary cholangitis; RIA, radioimmunoassay; SS, simple steatosis.

**Table 6:** Principal published studies evaluating the association of growth hormone deficiency (GHD) with NAFLD.

Author, Ref.	Method, Cohort, and Diagnostic Criteria	Findings
[143]	Longitudinal cohort study. 879 patients with hypothalamic or pituitary dysfunction. NAFLD diagnosis based on with imaging and liver enzyme alteration. Liver biopsy was performed in a subgroup of 10 NAFLD patients	NAFLD was found in 21 patients with metabolic syndrome-like phenotype (prevalence of 2.3%). The majority of biopsy-proven NAFLD patients exhibited advanced forms, i.e., cirrhosis and NASH with fibrosis
[144]	Cross-sectional retrospective study. 66 adults with hypopituitarism and GHD compared to 83 age-, gender-, and BMI-matched healthy controls. 19 patients received GH replacement therapy according to clinical recommendations. GHD diagnosis based on insulin tolerance test or GH releasing peptide-2 test. NAFLD diagnosis based on ultrasound. Liver biopsy was performed in 16 patients	NAFLD prevalence was significantly higher of 6.4-fold in GHD group compared to healthy controls, independently of obesity. Overweight and insulin resistance were more prevalent in GHD group with NAFLD. Histological NASH was found in 14 out of 16. GHRT was associated with reduction of liver enzyme and improvement of steatosis and fibrosis
[145]	Cross-sectional study. 18 adult patients with hypopituitarism, of these 13 with GHD. NAFLD diagnosis based on computer tomography (CT) (liver/spleen CT value <0.9)	Prevalence of hepatic steatosis higher in GHD subjects compared to hypopituitaric subjects without GHD, independently of BMI and triglycerides levels
[146]	Cross-sectional observational study. 34 Korean patients with hypopituitarism and 40 age- and sex-matched lean healthy controls. GHD defined as peak GH level of <3 ng/mL. NAFLD diagnosis based on ultrasound	Prevalence of fatty liver was significantly higher in men with hypopituitarism than controls. Among NAFLD patients with hypopituitarism, GH levels were lower and negatively correlated with degree of steatosis
[147]	Cross-sectional study. 28 patients with GHD and 24 age- and BMI-matched controls. 12 GHD patients evaluated longitudinally before and 6 months after start of GHRT. GHD diagnosis based on GH response <3 mg/L after glucagon stimulation. NAFLD defined as MRI-assessed intrahepatic lipid content (IHLC) > 5.6%	Although GHD patients exhibited higher visceral fat, the 2 groups presented similar liver enzyme levels and IHLC. GHRT was associated with reduction of subcutaneous and visceral adipose tissue and, in those with baseline high liver fat, with a positive trend in reduction of IHLC
[148]	Cross-sectional study. 22 adult patients with GHD and 44 age-, gender-, and BMI-matched healthy controls. 9 GHD patients received GHRT. GHD diagnosis based on GH levels <7.8 mU/L after glucagon stimulation test. NAFLD diagnosis based on IHLC >5.56% assessed with proton magnetic resonance spectroscopy (^1^H-MRS)	No significant difference in IHLC and prevalence of hepatic steatosis was observed in the two groups. In GHD patients receiving GHRT, no changes in IHLC were observed

Abbreviations: NAFLD, nonalcoholic fatty liver disease; NASH, nonalcoholic steatohepatitis; GH, growth hormone; GHD, growth hormone deficiency; BMI, body mass index; GHRT, growth hormone replacement therapy; IHCL, intrahepatic lipid content.

## Abbreviations

ACTH	adrenocorticotropic hormone
AGHD	adult growth hormone deficiency
AMPK	AMP-activated protein kinase
apo	apolipoprotein
BAAT index	body mass index, age, ALT, and triglyceride
BMI	body mass index
CCL3	C–C motif chemokine ligand 3
CI	confidence interval
ERK	extracellular signal-regulated kinase
FT4	free thyroxine
GH	growth hormone
GHD	growth hormone deficiency
GHRH	GH-releasing hormone
GHRLD	liver-specific deletion of GH receptor
HDL	high-density lipoprotein
hypertension	arterial hypertension
IGF-1	insulin-like growth factor-1
JAK-2	Janus kinase 2
JAK2L	liver-specific JAK2-deficient
JNK	c-Jun N-terminal kinase
LDL	low-density lipoprotein
LH	luteinizing hormone
MAPK	mitogen-activated protein kinase
MKK3	MAP kinase-activated protein kinase-3
MKK-4	MAP kinase-activated protein kinase-4
MRI	magnetic resonance imaging
MRS	magnetic resonance spectroscopy
NAFLD	nonalcoholic fatty liver disease
NASH	nonalcoholic steatohepatitis
OR	odds ratio
PCOS	polycystic ovary syndrome
PI3K	phosphoinositide 3-kinase
PKC	protein kinase C
PPARα	peroxisome proliferator-activated receptor-α
ROS	reactive oxygen species
SREBP-1c	sterol regulatory element-binding
STAT-5	signal transducer and activator of transcription 5
TNF-α	tumor necrosis factor-α
TSH	thyroid-stimulating hormone
VLDL	very low-density lipoprotein
